# A Rare Coincidence of Three Inherited Diseases in a Family with Cardiomyopathy and Multiple Extracardiac Abnormalities

**DOI:** 10.3390/ijms25147556

**Published:** 2024-07-10

**Authors:** Anna Bukaeva, Roman Myasnikov, Olga Kulikova, Alexey Meshkov, Anna Kiseleva, Anna Petukhova, Evgenia Zotova, Peter Sparber, Alexandra Ershova, Evgeniia Sotnikova, Maria Kudryavtseva, Anastasia Zharikova, Sergey Koretskiy, Elena Mershina, Vasily Ramensky, Marija Zaicenoka, Yuri Vyatkin, Alisa Muraveva, Alexandra Abisheva, Tatiana Nikityuk, Valentin Sinitsyn, Mikhail Divashuk, Elena Dadali, Maria Pokrovskaya, Oxana Drapkina

**Affiliations:** 1 National Medical Research Center for Therapy and Preventive Medicine, 101990 Moscow, Russia; andorom@yandex.ru (R.M.); olgakulikova2014@mail.ru (O.K.); meshkov@lipidclinic.ru (A.M.); sanyutabe@gmail.com (A.K.); anna.petukhova.96@gmail.com (A.P.); evgenia.d.zotova@gmail.com (E.Z.); alersh@mail.ru (A.E.); sotnikova.evgeniya@gmail.com (E.S.); kudryavtseva6041995@yandex.ru (M.K.); azharikova89@gmail.com (A.Z.); skoretskiy@gnicpm.ru (S.K.); ramensky@gmail.com (V.R.); vyatkin@gmail.com (Y.V.); alisa.fbb@gmail.com (A.M.); abisheva.alex@gmail.com (A.A.); tnikityuk@gnicpm.ru (T.N.); divashuk@gmail.com (M.D.); mpokrovskaya@gnicpm.ru (M.P.); odrapkina@gnicpm.ru (O.D.); 2 National Medical Research Center of Cardiology, 121552 Moscow, Russia; 3 Research Centre for Medical Genetics, 115522 Moscow, Russia; psparber93@gmail.com (P.S.); genclinic@yandex.ru (E.D.); 4 Department of General and Medical Genetics, Pirogov Russian National Research Medical University, 117997 Moscow, Russia; 5 Faculty of Bioengineering and Bioinformatics, Lomonosov Moscow State University, 119991 Moscow, Russia; 6 Medical Research and Educational Center, Lomonosov Moscow State University, 119991 Moscow, Russia; elena_mershina@mail.ru (E.M.); vsini@mail.ru (V.S.); 7 MSU Institute for Artificial Intelligence, Lomonosov Moscow State University, 119991 Moscow, Russia; 8 Moscow Center for Advanced Studies, 123592 Moscow, Russia; marija.zaicenoka@gmail.com; 9 All-Russia Research Institute of Agricultural Biotechnology, 127550 Moscow, Russia

**Keywords:** cardiomyopathy, left ventricular noncompaction, exome sequencing, functional study, *ALPK3*, *GATA3*, *WDR45*, personalized medicine, rare diseases, diagnostic odyssey

## Abstract

A genetic diagnosis of primary cardiomyopathies can be a long-unmet need in patients with complex phenotypes. We investigated a three-generation family with cardiomyopathy and various extracardiac abnormalities that had long sought a precise diagnosis. The 41-year-old proband had hypertrophic cardiomyopathy (HCM), left ventricular noncompaction, myocardial fibrosis, arrhythmias, and a short stature. His sister showed HCM, myocardial hypertrabeculation and fibrosis, sensorineural deafness, and congenital genitourinary malformations. Their father had left ventricular hypertrophy (LVH). The proband’s eldest daughter demonstrated developmental delay and seizures. We performed a clinical examination and whole-exome sequencing for all available family members. All patients with HCM/LVH shared a c.4411-2A>C variant in *ALPK3*, a recently known HCM-causative gene. Functional studies confirmed that this variant alters *ALPK3* canonical splicing. Due to extracardiac symptoms in the female patients, we continued the search and found two additional single-gene disorders. The proband’s sister had a p.Trp329Gly missense in *GATA3*, linked to hypoparathyroidism, sensorineural deafness, and renal dysplasia; his daughter had a p.Ser251del in *WDR45*, associated with beta-propeller protein-associated neurodegeneration. This unique case of three monogenic disorders in one family shows how a comprehensive approach with thorough phenotyping and extensive genetic testing of all symptomatic individuals provides precise diagnoses and appropriate follow-up, embodying the concept of personalized medicine. We also present the first example of a splicing functional study for *ALPK3* and describe the genotype–phenotype correlations in cardiomyopathy.

## 1. Introduction

Through extensive genetic studies over the past decades, the diagnostic yield of genetic testing in patients with inherited cardiomyopathies has reached 60% [1]. Most genotype-positive cases are associated with findings in sarcomeric protein genes with well-established gene–disease associations [2]. However, in the remaining cases, beyond this tier of well-known causative genes, a DNA diagnosis of primary cardiomyopathies may be very difficult due to complex or non-mendelian inheritance [3], an unknown/understudied causative gene [4], or the limitations of sequencing and variant interpretation techniques [5]. In the case of syndromic cardiomyopathies, the diagnosis is further complicated due to their prominent phenotypic heterogeneity, which includes extracardiac features. The currently known syndromes with left ventricular hypertrophy (LVH) (so-called “mimics of hypertrophic cardiomyopathy (HCM)”) [6] account for only 5–10% of diagnosed cases of HCM [7,8] and have highly variable clinical manifestations. For instance, RASopathies, the most well-known group of syndromes combining primary cardiomyopathies and facial/skeletal abnormalities, are themselves a highly heterogeneous entity with extensive phenotypic overlap [9,10]. Moreover, diverse extracardiac symptoms and multiorgan involvement may indicate the presence of two independent syndromes rather than syndromic cardiomyopathy, even though the probability of such a coincidence is usually considered to be low. Based on the above, in the case of cardiomyopathy with extracardiac involvement, even the proposition of a clinical diagnosis may be difficult and time-consuming, let alone a genetic diagnosis. In such cases, extended family anamnesis and cascade phenotype screening may be of particular importance, but these data are often unavailable [11].

Ongoing studies provide new insights into the strategy of the DNA diagnosis of cardiomyopathies, proposing new gene–disease correlations to evaluate and study. One of the remarkable innovations in the field is the rather recent discovery of the *ALPK3* gene as the cause of primary cardiomyopathy with facial and musculoskeletal abnormalities [12,13,14]. Available reports have hitherto shown high variability in the clinical manifestations of *ALPK3*-related cardiomyopathy, but the data on genotype–phenotype correlation are still scarce due to the limited number of published studies.

We report a case of successful genetic investigation of *ALPK3*-related cardiomyopathy with LVH, left ventricular noncompaction (LVNC), and extensive fibrosis in a large family with diverse dysmorphological features. The causative variant in *ALPK3* that we report here was previously mentioned in our LVNC cohort study [15], but the diagnostic odyssey in this family reached even further and revealed the complex genetic etiology of the observed lesions in the proband’s relatives. We would like to present this remarkable case as an illustration of a comprehensive, personalized approach to patients with unclear diagnoses.

## 2. Results

**Figure 1:**
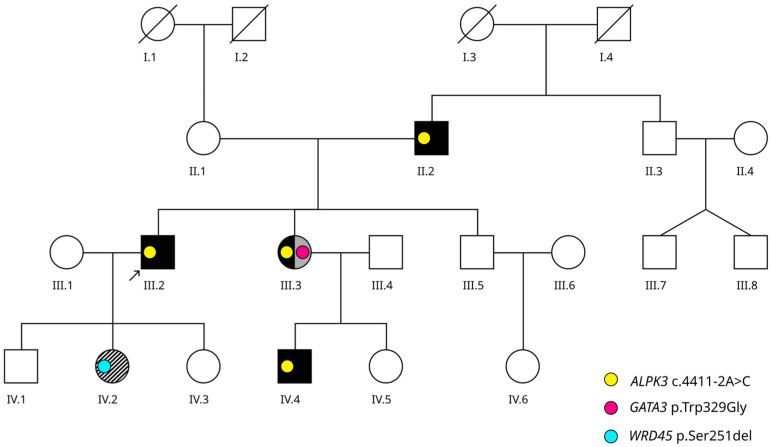
Pedigree. The proband is indicated by the arrow. Figures that represent the affected family members are colored in order to highlight the key phenotype features, as follows: the black color shows the cardiomyopathy phenotype; the grey color shows genitourinary malformations; and diagonal black–white shading shows epileptic seizures and developmental delay. Colored dots indicate distinct genetic variants present in the family.

The pedigree of the studied family is shown in Figure 1.

### 2.1. Clinical Characteristics of the Proband

**Figure 2:**
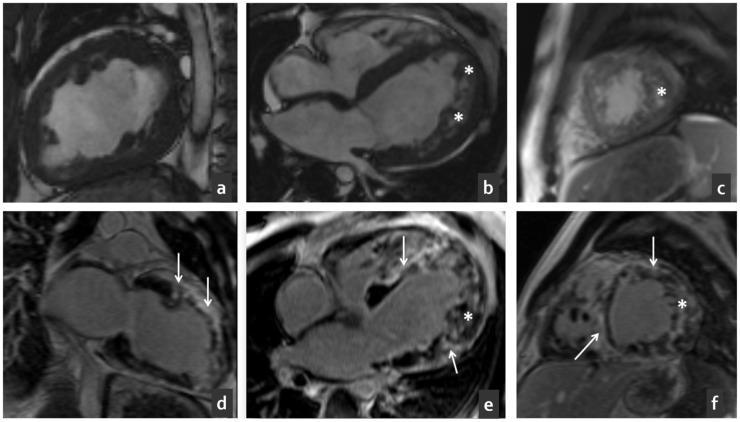
The proband’s CMR (III-2). (**a**–**c**) Cine mode, SSFP sequence; (**a**) long-axis two-chamber images, (**b**) long-axis four-chamber images, and (**c**) short-axis images. Asterisks mark the noncompacted layer up to 22 mm. (**d**–**f**) Delayed contrast enhancement, IR sequence with suppression of the signal from the myocardium. Extensive contrast accumulation of non-coronary origin in the front and lateral walls of LV and in the ventricular septum (marked with arrows). Streaks of contrast between the trabeculae of non-compacted myocardium.

The male proband (III-2, Figure 1), aged 41, was initially diagnosed with HCM at the age of 17 and prescribed beta blockers. In the following years, the patient was subject to regular control echocardiography, and no substantial progression of hypertrophy was observed during that period. At the age of 35, he developed a stroke and was hospitalized; subsequent echocardiography showed a decrease in the left ventricular ejection fraction (LVEF) to 42%, systolic pulmonary artery pressure (SPAP) of 2 mmHg, and myocardial hypertrabeculation. Coronary angiography did not reveal any pathological changes in the coronary arteries. On cardiovascular magnetic resonance (CMR) images (Figure 2), we observed left ventricular noncompaction (Petersen [16], Jacquier [17], Grothoff [18] criteria), late gadolinium enhancement (LGE) in the subendocardial regions and interventricular septum, and an LVEF of 35%. The CMR parameters of the heart were as follows: left atrial size = 5.5 cm, end diastolic diameter (EDD) = 6.1 cm, end systolic diameter (ESD) = 5.3 cm, and LV wall thickness = 16 mm. Particularly noteworthy were the severity of noncompaction (mass of noncompact layer (NCM) normalized to body surface area (BSA) = 40 g, NCM/total mass of LV (TM) ratio = 26%) and the abundance of fibrosis (in 14 of 17 segments of the LV).

**Figure 3:**
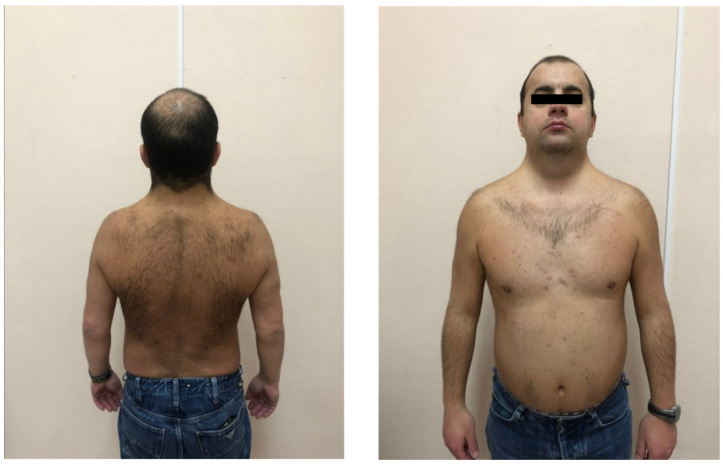
The proband of the studied family. Of note are the key dysmorphic features: short stature, low posterior hairline, and webbed neck.

The patient presented with short stature; a short, webbed neck; a low posterior hairline; low-set ears; a long philtrum; and brachydactyly (Figure 3). In the following years, the patient’s HF progressed, a left bundle branch block developed, and unstable ventricular tachycardia appeared, despite the ongoing therapy for HF. Later in 2017, the patient was implanted with a cardiac resynchronization device, but it did not lead to any improvement of EF and heart dimensions. The patient was in a stable condition for several years but deteriorated in 2022. Due to the inefficiency of drug therapy, the patient underwent orthotopic heart transplantation in August 2022. In the early postoperative period, the patient died of kidney failure. A histological examination of the explanted heart confirmed the morphology of non-obstructive hypertrophic cardiomyopathy.

### 2.2. Phenotypic Cascade Screening of the Relatives

**Figure 4:**
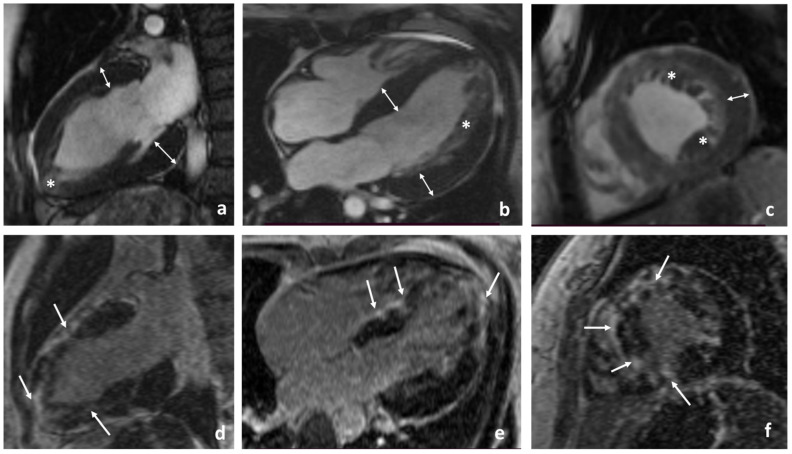
Cardiac MRI of the proband’s sister (III-3). (**a**–**c**) Cine mode, SSFP sequence; (**a**) long-axis two-chamber images, (**b**) long-axis four-chamber images, and (**c**) short-axis images. Asterisks mark the noncompacted layer in the apical region and throughout the walls of LV; arrows show the total hypertrophy of all the LV walls. (**d**–**f**) Delayed contrast enhancement; IR sequence with suppression of the signal from the myocardium. Arrows show extended intramyocardial fibrosis of a non-coronary origin in the LV apex, interventricular septum, and anterior and lateral LV walls.

The proband’s sister (III-3, Figure 1), aged 46, was diagnosed with sensorineural hearing loss at the age of 12. At the age of 13, routine ultrasound imaging revealed the absence of one kidney. Later, at the age of 20, during pregnancy, she was found to have uterus didelphys (doubled uterus). At the same time, she began to notice shortness of breath during physical exertion and periodic heart pain. A cardiovascular examination showed myocardial hypertrophy; consequently, she was prescribed metoprolol. At the age of 35, she experienced episodes of palpitations with darkening in the eyes and underwent an instrumental examination. Echocardiography revealed asymmetric myocardial hypertrophy (LV thickness 17-26-16 mm) and an LVEF of 75%. Transesophageal stimulation induced an unstable paroxysm of fibrillation–atrial flutter. She was prescribed metoprolol in combination with perindopril and eplerenone. At the age of 42, she was admitted to the Center for Therapy and Preventive Medicine. Her ECHO showed left atrial size = 39 mm, EDD = 49 mm, LV thickness = 17–26 mm, LVEF = 50%, SPAP = 35 mmHg, moderate diffuse myocardial hypokinesis of the LV, multiple foci of intramural myocardial fibrosis in the LV, and increased trabeculation of the myocardium. On CMR images (Figure 4), prominent noncompaction was confirmed, NCM normalized to BSA was 23.5 g, and the NCM/TM ratio was 16%. We also observed myocardial fibrosis in 9 of 17 segments.

Holter ECG (on 100 mg/day metoprolol therapy) did not detect any ventricular arrhythmias. At the age of 43, follow-up ECHO showed a decrease in LVEF to 47%, diffuse hypokinesis, and diastolic dysfunction type 2, and follow-up Holter ECG showed unstable episodes of VT of 6 complexes with a heart rate of 180 per minute. According to the HCM Risk-SCD scale [19], her risk of sudden cardiac death was 4.9%. Consequently, she was implanted with a two-chamber cardioverter defibrillator. In August 2022, following the death of her younger brother (III-2), she had a transitory ischemic attack due to emotional stress; computer tomography of the brain did not reveal any lesions. Her latest cardiological examination showed a further decrease in LVEF and a progression of HF.

The proband’s father (II-2, Figure 1), aged 68, has hypertension, LVH (LV thickness of 13 mm), and an LVEF of 53%.

The proband’s eldest daughter (IV-2, Figure 1), aged 12, had been previously diagnosed with “atypical autistic spectrum disorder (ASD)” due to absence epileptic seizures, psychomotor delay, and speech delay. Her epilepsy is currently in remission due to suxilep treatment. The girl exhibits emotional lability, aggressiveness, dysarthria, and a moderate intellectual disability. She is educated at a correctional school. During her physical examination, we noticed a number of facial stigmas, such as a short, thick neck; a coarse face with a narrow forehead; a long philtrum; and synophrys. Her ECHO showed no signs of cardiomyopathy but revealed excessive trabeculation.

The proband’s nephew (IV-4, Figure 1), aged 25, has no complaints. On his ECHO, we observed a moderate dilation of LV (EDV = 154 mL), an LVEF of 50%, LV thickness of 11 mm, and myocardial hypertrabeculation.

The proband’s son (IV-1, aged 9) is healthy. According to ECHO, his LVEF is 68%, and LV thickness is 5 mm. The proband’s younger daughter (IV-3, aged 7) is also healthy; on her ECHO, her EF was 71%, and LV thickness was 5 mm. No signs of noncompact myocardium were observed in patients IV-1 and IV-3.

The proband’s brother (III-5, aged 35) has no complaints. On his ECHO, his LVEF was 66%, and LV thickness was 10 mm, without any signs of LVNC. The proband’s wife (III-1, aged 42) is also apparently healthy.

The aforementioned clinical data are summarized in Supplementary Table S1.

### 2.3. Genetic Testing Results

Genetic testing of the proband revealed a NM_020778.5:c.4411-2A>C variant in the canonical acceptor splice site of exon 11 in the *ALPK3* gene. Recently, heterozygous truncating variants in *ALPK3* (*ALPK3*tv) have been identified as the cause of adult-onset HCM [13]. Based on sequence properties of *ALPK3*, we have suggested that the variant is most likely affecting splicing. Cascade screening in the family showed the presence of the *ALPK3* variant in the proband’s affected sister (III-3), her son (IV-4), and also in the affected siblings’ father (II-1).

Additionally, in the proband’s sister, we found a novel variant, NM_001002295.2:c.985T>G, leading to the missense change p.Trp329Gly in *GATA3*, the gene responsible for the rare syndrome “hypoparathyroidism, sensorineural deafness, and renal dysplasia” with multiple causal pathogenic variants reported [20]. We considered this variant pathogenic according to the ACMG guidelines [21] (PM2, PM1, PS2, PP3, PP4). Moreover, in the proband’s eldest daughter, affected with developmental delay and facial abnormalities, we found an in-frame deletion NM_007075.3:c.752_754delCCT (p.Ser251del) in the *WDR45* gene, previously described in connection to beta-propeller protein-associated neurodegeneration—a rare form of neurodegeneration with brain iron accumulation [22,23]. Based on the ACMG guidelines, we classified this variant as likely pathogenic (PM2, PM4, PS2, PP5). All genetic findings and their segregation in the family are summarized in Supplementary Table S1. In addition, detailed information on the reported genetic variants is displayed in Supplementary Table S2.

### 2.4. Functional Analysis

To confirm that the c.4411-2A>C variant in the *ALPK3* gene indeed leads to splicing change and to establish the exact nature of this alteration, a functional analysis of the variant was performed using a minigene assay. The minigene assay was used because, according to the GTEx portal, the *ALPK3* gene is not expressed in accessible tissues.

**Figure 5:**
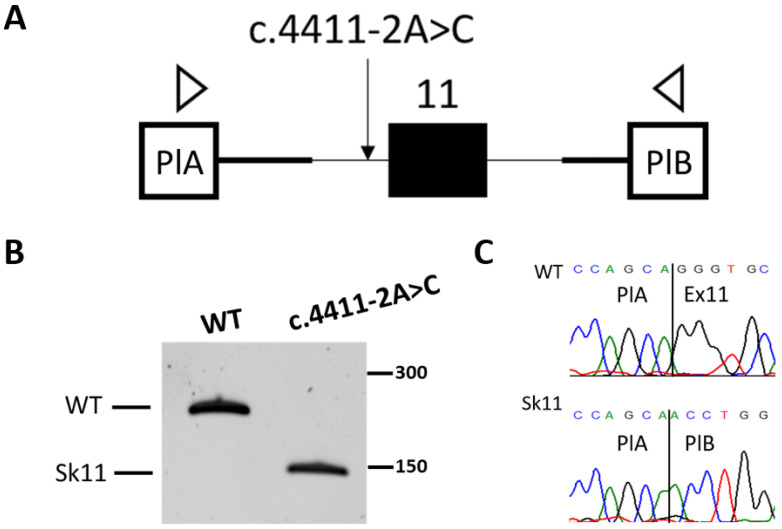
Functional analysis of the c.4411-2A>C variant in the *ALPK3* gene: (**A**) scheme of the minigene plasmid with the c.4411-2A>C variant. The arrows indicate the location of the primers used for RT-PCR; (**B**) plasmid-specific RT-PCR products in denaturing PAGE; and (**C**) Sanger sequencing of WT and mutant isoforms. PlA and PlB—plasmid exons. Sk11—skipping of exon 11.

A wild-type (WT) plasmid and a plasmid carrying the c.4411-2A>C variant were transfected separately into HEK293T cells, and 48 h post-transfection, total RNA was extracted, and RT-PCR was performed. RT-PCR analysis demonstrated that the c.4411-2A>C variant leads to disruption of the acceptor splicing site and complete skipping of exon 11 (Figure 5).

The skipping of exon 11 leads to a frameshift (p.(Gly1471Aspfs*39)), which likely activates the nonsense-mediated decay (NMD) pathway [24] and hence leads to the complete absence of the protein from one allele. However, as some transcripts with premature termination codons may escape NMD [25], a bioinformatic analysis of the possible truncated protein was performed.

If translated, the skipping of exon 11 would lead to the formation of an ALPK3 protein with an altered C-terminus lacking the alpha-type protein kinase domain, where many missense and loss-of-function (LoF) variants were previously described in patients with hypertrophic cardiomyopathy [13,26,27].

Based on the results of the minigene assay, the c.4411-2A>C variant in the *ALPK3* gene was classified as pathogenic according to the ACMG guidelines (PM2, PVS1, PS3).

## 3. Discussion

The proband of the studied family was admitted to our center because of hypertrophic cardiomyopathy, accompanied by left ventricular noncompaction and dysmorphic features. According to the patient’s anamnesis and medical documentation, he had undergone genetic testing for inherited cardiomyopathies and Noonan-like disorders several times before in different commercial institutions, but to no avail. Given the atypical clinical appearance of the proband, the presence of extracardiac symptoms, and previous experience with negative genetic testing, we chose whole-exome investigation as the diagnostic approach in this case. As a result, we revealed the previously unreported alteration of the canonical splice site in *ALPK3*.

The first paper linking the *ALPK3* gene to cardiomyopathies was published in 2016 [12] and focused on biallelic truncating variants in this gene, considering them to be the cause of severe autosomal recessive (AR) cardiomyopathy with neonatal onset. The following publications highlighted some distinct features specific to *ALPK3*-dependent AR cardiomyopathy, such as an unusual progression of neonatal dilated cardiomyopathy to HCM [28,29] and various facial and musculoskeletal dysmorphisms [14,29,30]. Along with this, some of the reports showed that heterozygous carriers of *ALPK3*tv may also be affected by cardiomyopathy [30,31]. Further studies and the accumulation of the data established a robust association between monoallelic (heterozygous) *ALPK3*tv and HCM inherited in an autosomal dominant (AD) way [13,27,32]. For now, the existing evidence is sufficient to definitely speak of the causative role of *ALPK3* variants in HCM both in AR and AD modes of inheritance, with a general understanding of the AR form as severe early-onset and the AD form as adult- or late-onset, without significant extracardiac involvement [33].

However, due to the relative novelty of the topic, the amount of information on the phenotypes of *ALPK3*tv carriers, especially for AD inheritance, is still small, and the clinical features described so far are very diverse, leaving us far from understanding the full landscape of genotype–phenotype correlations. For instance, the age of onset varies widely, with a number of biallelic cases manifesting in the first 2–3 decades of life [14,34]. The matter is further complicated when speaking of the extracardiac features of *ALPK3*-dependent cardiomyopathy. The studies published to date show a vast spectrum of extracardiac alterations in patients with *ALPK3* variants, including various dysmorphic craniofacial features [29,30,35], skeletal abnormalities [27,30], joint contractures [36], myopathic features [13,37], and developmental delay [14,36], including speech delay [27]. Though sometimes *ALPK3*-related cardiomyopathy is referred to as “Noonan-like” [35], published observations to date demonstrate that the full spectrum of extracardiac manifestations is much wider than in Noonan-like disorders. All of the above testifies to the highly variable expressivity and penetrance of *ALPK3* pathogenic variants.

The variant that we found was proven to be pathogenic by the functional study. Cascade screening of the family showed the presence of HCM/LVH in three of four carriers of this variant. The fourth carrier showed a tendency to LV dilation and lowering of myocardium contractility, which we interpret as cardiomyopathy based on family anamnesis. Thus, in this case, the penetrance of the finding in *ALPK3* reaches 100%, although the expressivity of alterations is highly variable within the family.

The clinical appearance of the proband and his sister is remarkable for the combination of moderate hypertrophy and prominent myocardial noncompaction, extended fibrosis, and the tendency to dilation of the heart chambers. Over the years of follow-up, we observed a steadily progressive decrease in global myocardial contractility with signs of restriction. Moreover, of note were the life-threatening alterations of heart rhythm, which required the implantation of cardiac resynchronization devices. Overall, the cardiomyopathy phenotype of our patients is mainly consistent with the published data. However, several features were not quite typical for the previous observations; for instance, the onset of HCM in the proband occurred in the second decade of life, which is earlier than in most reported monoallelic cases. Moreover, the extracardiac features observed in this family attracted further attention.

Particularly, the sensorineural hearing loss and genitourinary malformations in the proband’s sister were unlikely to be explained by *ALPK3* haploinsufficiency. Although one of the previously reported patients was described as “presenting at age 24 with renal disease” [34], and one more case describes a heterozygous *ALPK3* carrier with independently inherited polycystic kidney disease [14], it did not seem to be a convincing genotype–phenotype correlation. The phenotype of the proband’s daughter was even more confusing, since her facial features and developmental delay partially fitted the disorders described in biallelic *ALPK3*, but cascade genetic screening was negative. Given these discrepancies, we studied the whole exome in all affected family members and discovered two new findings correlating with independent inherited syndromes.

In the proband’s sister, we found a p.Trp329Gly missense in *GATA3*, the gene responsible for the rare syndrome “hypoparathyroidism, sensorineural deafness, and renal dysplasia” (HDR). Her parents (II-1 and II-2) lacked this variant. The HDR syndrome is highly clinically variable, with renal morphological abnormalities occurring in 61% of cases and sensorineural deafness occurring in 92.7% [38]. Uterus didelphys has also been reported as a *GATA3*-associated disease [39]. The finding in our patient occurs in the second zinc finger (ZnF2) domain, and the missense variants in HDR syndrome are known to cluster in the ZnF domains [38]. Sadly, we were not able to make any functional analysis for this missense variant, but we suggest its causative role in our patient since the symptoms are highly specific. Thus, we classify the *GATA3* p.Trp329Gly substitution as pathogenic with the following ACMG pathogenicity criteria: PM2, PM1, PS2, PP3, and PP4.

In the proband’s eldest daughter, presenting with an ASD-like phenotype and facial stigmas, we found an in-frame deletion p.Ser251del in the *WDR45* gene, absent in her parents (III-1 and III-2). This deletion was previously reported in a patient with beta-propeller protein-associated neurodegeneration (BPAN) [22]. BPAN is one of the most frequent forms of neurodegeneration with brain iron accumulation (NBIA) [40]. Its estimated worldwide prevalence is two to three per million individuals [41]. This is an X-linked dominant disorder; the affected patients present in childhood with developmental delay, epilepsy, and dystonia and develop Parkinson-like clinical features and dementia later in adulthood. The clinical manifestations of BPAN are not highly specific by itself but are consistent with the neurological phenotype of patient IV-2. We classify this in-frame deletion as likely pathogenic (PM2, PM4, PS2, PP5). Unfortunately, the link between our finding in *WDR45* and the girl’s diagnosis is our speculation for now, since we were not able to perform a brain MRI and confirm the presence of iron accumulation.

The occurrence of three independent causal variants within a single family is an exceedingly rare phenomenon, which may also be a heritable trait. Despite the absence of a familial cancer history, the pattern of de novo effects following paternal lineage may suggest the potential role of genes that maintain genome stability during spermatogenesis [42]. However, this hypothesis is hard to prove and requires additional investigation outside of the scope of this clinical case.

### Limitations of this Study

This study was mainly focused on the cardiomyopathy aspects of the family since it is the main field of expertise in our team. The severity of cardiomyopathy in the proband and his sister is not fully consistent with the known genotype–phenotype correlations and may be possibly caused by the presence of an undiscovered structural variation in the second allele of *ALPK3*, which we would not be able to find by exome sequencing. Also, we could not confirm the pathogenic role of the genetic findings in the proband’s sister and eldest daughter. As for the latter, we cannot fully exclude the possibility of maternal inheritance of the *WDR45* in-frame deletion due to mosaicism or skewed X inactivation. More than that, the detailed investigation and further management of the *WDR45*-dependent disorder are beyond the scope of this team of authors. Currently, we are redirecting the girl to expert neurologists for further investigation and follow-up.

Notably, the coincidence of three distinct inherited diseases in the same family is itself an outstanding and almost unbelievable case that needs maximum possible confirmation. However, such cases, despite being extremely rare, may still occur and illustrate the importance of “dulling Occam’s razor” [43] when observing complex, unexplained phenotypes.

## 4. Materials and Methods

### 4.1. Clinical Investigation

Three generations of the family with LVNC (13 people in total) were admitted to the National Research Center for Therapy and Preventive Medicine (Moscow, Russia). A clinical cardiological investigation was performed in accordance with current European recommendations for the management of cardiomyopathies [7] and included blood tests, standard 12-lead and Holter monitoring electrocardiograms (ECG), echocardiography (ECHO), and cardiac magnetic resonance (CMR) imaging. This study was performed according to the principles of the Declaration of Helsinki and approved by the Institutional Review Boards of the Medical Research Center for Therapy and Preventive Medicine (Moscow, Russia). Every participant and/or their legal representative gave their written informed consent to be involved in this study.

### 4.2. Whole-Exome Sequencing and Bioinformatic Analysis

The molecular genetic investigation was carried out as we previously detailed [44]. We performed whole-exome sequencing for the proband, his parents, siblings, spouse, and children—a total of nine persons. For the cascade screening of the remaining family members, we used Sanger sequencing.

For the whole-exome sequencing, we isolated DNA from whole blood samples using the QIAamp DNA Blood Mini Kit (Qiagen, Hilden, Germany), then prepared IDT-Illumina TruSeq DNA exome libraries, and performed sequencing on NextSeq 550 (Illumina, San Diego, CA, USA) in accordance with the manufacturer’s protocols.

Sequencing reads were aligned to the reference genome (GRCh38) using bwa-mem v2-2.2.1 [45]; single nucleotide variants were called with the GATK 4.2.2.0 HaplotypeCaller [46] and annotated using the Ensembl Variant Effect Predictor v104 [47]. Potentially clinically relevant findings were interpreted in accordance with current international guidelines [21]. Candidate causal variants were validated by Sanger sequencing on the Applied Biosystems 3500 Genetic Analyzer (Thermo Fisher Scientific, Waltham, MA, USA) using the ABI PRISM BigDye Terminator reagent kit v. 3.1 (Thermo Fisher Scientific, Waltham, MA, USA).

### 4.3. Minigene Splicing Assay

The minigene splicing assay was performed as previously described [48]. Briefly, exon 11 of the ALPK3 gene, along with a minimum of 250 bp introns 10 and 11, were amplified from the proband’s genomic DNA using Q5 High-Fidelity DNA Polymerase (New England Biolabs, Ipswich, MA, USA). The PCR product was cloned into a pSpl3-Flu2 plasmid vector using Gibson Assembly^®^ Master Mix (NEB, USA). Sanger sequencing was used for the selection of clones carrying wild-type (WT) and the c.4411-2A>C variant. WT and mutant plasmids were separately transfected into HEK293T cells using Lipofectamine 3000 (Thermo Fisher Scientific, USA) according to the manufacturer’s protocol. Total RNA was isolated from the cells 48 h after transfection using the standard Trizol-based method, treated with DNAse I (Thermo Scientific Scientific, USA), and reverse transcribed using the ImProm-II™ Reverse Transcription System (Promega, Madison, WI, USA). Plasmid-specific primers were used in the PCR for the detection of possible splicing alterations. PCR products were analyzed by denaturing PAGE with 8M urea with further Sanger sequencing.

## 5. Conclusions

This outstanding clinical case demonstrates an extremely rare combination of three independent monogenic disorders in one family that had a long-term follow-up for HCM. The full explanation of all the abnormalities not only in the proband but also in his relatives became possible through a profound clinical investigation and extensive genetic testing that covered all known gene–phenotype correlations and was not limited to cardiomyopathies. We consider this approach, despite its labor- and time-consuming nature, good practice in studying complex, unusual clinical cases.

We also hope that our work contributes to the growing body of information on genotype–phenotype correlations in *ALPK3*tv carriers and disease mechanisms, particularly considering the association between *ALPK3*tv and LVNC. We suggest that genetic testing in patients with LVNC should include the *ALPK3* gene.

## Data Availability

The data presented in this study are available upon request from the corresponding author.

## Supplementary Materials

The following supporting information can be downloaded at https://www.mdpi.com/article/10.3390/ijms25147556/s1.
